# Commentary: The role of the Chief Health Strategist in community health improvement: a MAPP 2.0 counterproposal

**DOI:** 10.3389/fpubh.2025.1711850

**Published:** 2025-12-15

**Authors:** Anna Clayton, Bianca Lawrence, Tahlia Gousse

**Affiliations:** National Association of County and City Health Officials, Washington DC, United States

**Keywords:** community health improvement, health equity, community power building, cross-sector partnerships, local public health system, systems change

## Introduction

This Commentary is in response to Carman and Pendergrass's (2025) counterproposal to the National Association of County and City Health Officials' (NACCHO's) Mobilizing for Action through Planning and Partnerships (MAPP) 2.0 framework. MAPP was developed in 2001 as a community-driven strategic planning framework for public health systems change. The updated framework, MAPP 2.0, was released in 2023 based on national evaluation findings and input from local health departments and cross-sector partners. According to NACCHO's 2022 Profile of Local Health Departments (LHDs), more than half of LHDs who completed a community health assessment or community health improvement plan in the last 5 years used MAPP (1.0 and 2.0) (1).

The authors suggest that MAPP 2.0 be “reorganized as guidelines and suggestions for the Chief Health Strategist to use while working with the community” to maximize the local health official's strengths in strategic relationship building and community engagement. We describe why it is critical for MAPP to remain a community-owned process to build community power and collective leadership that are necessary for systems change. We propose that the local health department instead adopt the “Community Health Strategist” role, supporting local organizations to build accountability for creating a healthier community.

## Community power building and strategic collaboration through MAPP 2.0

The goal of MAPP 2.0 is to achieve health equity through a three-phase process that builds partnerships and community engagement (Phase I), conducts a comprehensive assessment of community health (Phase II), and facilitates action across the local public health system (Phase III). Carmen and Pendergrass suggest a counterproposal in which MAPP 2.0 activities fall into the role of the Chief Heath Strategist (2). For example, they suggest that partnership development activities, such as those in Phase I and the Community Partner Assessment, be reorganized “as part of [the Chief Health Strategist's] ongoing role in community networking…potentially reducing the burden on those partners to invest significant time answering organizational questions”. However, reducing partners' investment in relationship building, and centering MAPP leadership, may undermine MAPP 2.0's success.

Building healthier communities that achieve equitable outcomes requires systems thinking, strategic collaboration, and rebalanced power. MAPP 2.0's Foundational Principles include “strategic collaboration and alignment”, meaning the local public health system works together to advance equity, and “community power,” which emphasizes that the process is guided by the decisions of community members most impacted by inequities (3). Recognizing and addressing power imbalances, particularly the traditional dominance of institutions of authority like local governmental public health, is central to MAPP 2.0. It provides tools including the *Spectrum of Community Engagement to Ownership*, which outlines various levels of community decision-making power, to guide movement toward community ownership (4).

Local ownership over public health initiatives has been shown to achieve policy and system-level change, as well as improvements in disease prevention and severity, care services, and the environment (5). For example, Rhode Island has funded “10 ‘place-based' public health initiatives in geographically defined ‘health equity zones”' (6) since 2015 to address priorities such as food and nutrition, tobacco control policy, environmental health, housing, and education, and has reported successes in passing local ordinances prohibiting smoking in public parks, identifying socioeconomic factors driving poor health outcomes, and decreasing childhood lead poisoning (7). Furthermore, local ownership has value “in and of itself” because it builds community members' feelings of confidence and empowerment (6).

## The Community Health Strategist

For MAPP 2.0 to be successful, the local health department might instead adopt the “Community Health Strategist” model. This represents the entire local health department working as “a team leading a broader coalition of community partners from behind, rather than a single “chief” in front of a pack” (8). This model is preferred by many local health officials to achieve Public Health 3.0 and requires the public health workforce to strengthen skills in systems thinking and strategic collaboration and allows for greater community ownership over efforts to strengthen the local public health system.

The activities in Phase I and Phase 2 of MAPP 2.0 allow the health department to act as a Community Health Strategist, transferring power to the community and supporting partners to build relationships. In Phase I, the Stakeholder and Power Analysis reveals community members and groups impacted by inequities who should be engaged, and a visioning activity builds alignment across community shareholders. MAPP 2.0 was redesigned in ideals of collective impact (9) “collective leadership distributed among various roles,” and guides development of a Steering Committee to ensure the process is informed by diverse perspectives. In Phase II, the Community Partner Assessment engages organizations in a survey and a series of discussions to build trust and understand how they can advance equity together.

These activities build on one another to establish new relationships, strengthen care networks, prompt resource sharing, and address gaps in services, which contribute to a more effective local public health system. Filtering them through a singular Chief Health Strategist would undermine their value, could increase risk of setbacks due to turnover, and may weaken partners' accountability for systems change, as they may view the local health department, or official, as the leading authority. Without building a strong foundation of relationships, shared power and accountability, it would be difficult for the community to take collective action in the final Phase.

## Discussion

The authors suggest that the Chief Health Strategist lead MAPP 2.0 activities to maximize their ability to build trust, galvanize community member participation, and lead strategic thinking. However, for MAPP 2.0 to be successful, it must be community-driven throughout all phases. The activities build collective ownership to address power imbalances, create shared accountability over implementation, and benefit the local public health system overall. The modified proposal may undermine relationship building, shared leadership and accountability that are essential to community health improvement. We commend the authors for bringing forth the importance of the role of the Chief Health Strategist but highlight that the individual local health official fulfilling that role and assuming ownership over critical activities would not support the Foundational Principles or achieve the desired outcomes of MAPP 2.0.
